# Phytochemical Analysis, Antimutagenic and Antiviral Activity of *Moringa oleifera* L. Leaf Infusion: In Vitro and In Silico Studies

**DOI:** 10.3390/molecules27134017

**Published:** 2022-06-22

**Authors:** Ika Rahayu, Kris Herawan Timotius

**Affiliations:** 1 Biochemistry Department, Faculty of Medicine and Health Sciences, Universitas Kristen Krida Wacana (UKRIDA), Jakarta 11510, Indonesia; ika.rahayu@ukrida.ac.id; 2 Research Center for Jamu and Herbal Medicine, Universitas Kristen Krida Wacana (UKRIDA), Jakarta 11510, Indonesia

**Keywords:** AutoDock, COVID-19, SARS-CoV-2, flavonoids, anti-DNA damage, antioxidant, infusion, *kelor*

## Abstract

*Moringa oleifera (M. oleifera)* leaves are rich in nutrients and antioxidant compounds that can be consumed to prevent and overcome malnutrition. The water infusion of its leaf is the easiest way to prepare the herbal drink. So far, no information is available on the antioxidant, antimutagenic, and antivirus capacities of this infusion. This study aimed to determine the composition of the bioactive compounds in *M. oleifera* leaf infusion, measuring for antioxidant and antimutagenic activity, and evaluating any ability to inhibit the SARS-CoV-2 main protease (Mpro). The first two objectives were carried out in vitro. The third objective was carried out in silico. The phytochemical analysis of *M. oleifera* leaf infusion was carried out using liquid chromatography-mass spectrometry (LC-MS). Antioxidant activity was measured as a factor of the presence of the free radical 2,2-diphenyl-1-picrylhydrazyl (DPPH). The antimutagenicity of *M. oleifera* leaf powder infusion was measured using the plasmid pBR322 (treated free radical). The interaction between bioactive compounds and Mpro of SARS-CoV-2 was analyzed via molecular docking. The totals of phenolic compound and flavonoid compound from *M. oleifera* leaf infusion were 1.780 ± 5.00 µg gallic acid equivalent/g (µg GAE/g) and 322.91 ± 0.98 µg quercetin equivalent/g (µg QE/g), respectively. The five main bioactive compounds involved in the infusion were detected by LC-MS. Three of these were flavonoid glucosides, namely quercetin 3-O-glucoside, kaempferol 3-O-neohesperidoside, and kaempferol 3-α-L-dirhamnosyl-(1→4)-β-D-glucopyranoside. The other two compounds were undulatoside A, which belongs to chromone-derived flavonoids, and gentiatibetine, which belongs to alkaloids. The antioxidant activity of *M. oleifera* leaf infusion was IC50 8.19 ± 0.005 µg/mL, which is stronger than the standard butylated hydroxytoluene (BHT) IC50 11.60 ± 0.30 µg/mL. The infusion has an antimutagenic effect and therefore protects against deoxyribonucleic acid (DNA) damage. In silico studies showed that the five main bioactive compounds have an antiviral capacity. There were strong energy bonds between Mpro molecules and gentiatibetine, quercetin, undulatoside A, kaempferol 3-o-neohesperidoside, and quercetin 3-O-glucoside. Their binding energy values are −5.1, −7.5, −7.7, −5.7, and −8.2 kcal/mol, respectively. Their antioxidant activity, ability to maintain DNA integrity, and antimutagenic properties were more potent than the positive controls. It can be concluded that leaf infusion of *M. oleifera* does provide a promising herbal drink with good antioxidant, antimutagenic, and antivirus capacities.

## 1. Introduction

*Moringa oleifera (M. oleifera)*, a family member of *Moringaceae* with the Indonesian common name Kelor, has good nutritional value and has been used to prevent malnutrition. *M. oleifera* leaves, pods, and seeds are known as high nutrition food. The most widely form consumed as a vegetable is *M. oleifera* leaves, which contain vitamin C, vitamin A, calcium, protein, potassium, and iron [1].

*M. oleifera* leaves are traditionally used as a supplement for increasing milk production in nursing mothers and as a supplement for children [2]. The bioactive compounds have many biological activities, such as antioxidant, anti-hyperglycemic, anti-inflammatory, anti-diabetic, antimicrobial, and anticancer activity [3]. These biological activities are related to their high antioxidant activity, which helps reduce free radical activity in the body, which in turn causes oxidative stress, triggering the development of various chronic and degenerative diseases [4,5]. These free radicals are produced by normal cell metabolism in situ or from environmental factors, such as pollution, cigarette smoke, radiation, and harmful drugs [6].

Free radicals are one of the many essential factors that cause DNA damage, mutations, or epigenetic disturbances. This damage occurs in the initiation phase of the carcinogenesis stage and leads to chronic degenerative diseases, such as atherosclerosis, cardiovascular disease, and neuro-ophthalmic disorders [7]. *M. oleifera* leaves have good nutritional value and are also thought to be able to maintain the integrity of DNA or genomes. The phytochemical content and antioxidant activity of *M. oleifera* leaves can nurture genome integrity. *M. oleifera* leaves might defend the homeostasis of DNA synthesis and repair, thus preventing the DNA damage caused by oxidative stress and methylation [8].

Recently, during the COVID-19 pandemic, a number of traditional medicinal ingredients were promoted as drugs to prevent or restore the COVID-19 disease, such as 1,8-Cineol essential oil, several other essential oils, and *Andrographis paniculata* extract [9,10,11]. Considering that *M. oleifera* leaves are widely consumed as vegetables that have a number of health benefits, an in-silico analysis was conducted to explore the possible interactions of the bioactive compounds contained in *M. oleifera* leaves and the main protease (Mpro) of the SARS-CoV-2 protein. Understanding these interactions can help us to gain knowledge on whether *M. oleifera* leaf infusion has the capacity for anti-SARS-CoV-2 treatment.

Therefore, this study was conducted with the aim of analyzing the composition of bioactive compounds in the aqueous extract or infusion of *M. oleifera* leaves using liquid chromatography-mass spectrometry (LC-MS), measuring their antioxidant activity with the 2,2-diphenyl-1-picrylhydrazyl (DPPH) test, and their ability to prevent DNA-damage using the plasmid pBR322. In addition to in vitro analysis, an in silico analysis was also conducted to determine the antiviral power of *M. oleifera* leaves.

## 2. Results and Discussion

**Figure 1:**
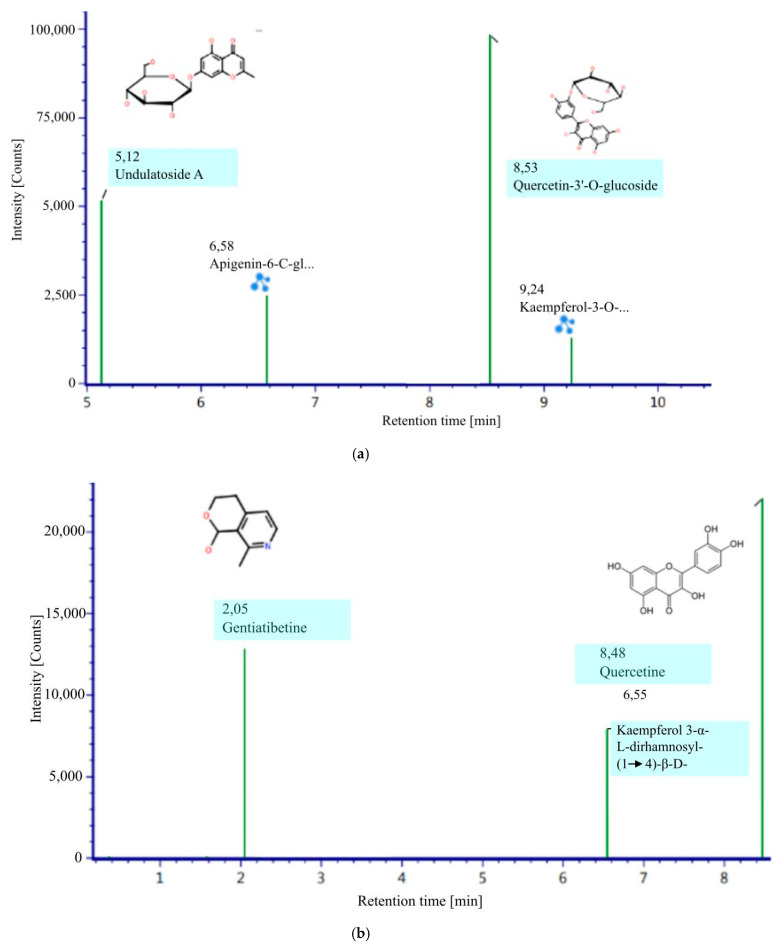
Bioactive compound negative ESI (**a**) and positive ESI (**b**).

LC-MS is an effective tool for identifying and characterizing phenolic and flavonoid compounds [12]. Identification and characterization of compounds were carried out via the comparison of the retention times (RT). Mass spectrometry (MS) data were obtained from both ionization modes, namely negative and positive electrons (ESI−/ESI+). Table 1 and Figure 1 show all of the compounds tentatively identified from the *M. oleifera* infusion in positive and negative ionization modes. One alkaloid compound and six flavonoid compounds (three free and three flavonoid glycosides) can be detected in *M. oleifera* leaf infusion. Additionally, undulatoside A, a chromone, was also detected.

The detected alkaloid compound was gentiatibetine. This alkaloid compound was first reported as an alkaloid found in *M. oleifera* leaf infusion. *M. oleifera* leaves are a good source of gentiatibetine. Gentiatibetine has anticonvulsant and brain-protective effects [13].

*M. oleifera* leaf infusion contains three known flavonoids: apigenin, quercetin, and kaempferol, either in a free state or as glycosides. They have been reported by several researchers [14,15]. They are known as flavonoids which have anticancer properties [16,17,18,19]. These properties are supported by a high antioxidant capacity [20,21] and antimutagenic agency [22]. Apigenin, quercetin, and kaempferol provide DNA protection from H_2_O_2_-induced damage [17,23,24,25,26]. There are various other flavonoids that have been reported in various studies, for example epicatechin, scopoletin, chlorogenic acid, rutin, and procyanidin [14,27]. This difference is mainly caused by the solvent used for extraction.

Undulatoside A is also a compound that has been detected for the first time in *M. oleifera* leaf infusion. This compound is also found in *Dryopteris fragrans* [13], *Eucalyptus* [28], *Conidium monnieri* [29], *Anchusa undulata* [30], *Evolvulus linarioides* [31], and *Knoxia corymbosa* [32]. The bioactivity of these compounds may include antimicrobial [30], anti-inflammatory properties [13,31,33] and immunomodulatory activity [32].

The total phenolic content (TPC) of *M. oleifera* leaf infusion was higher than the flavonoid content (TFC). The results of the study on TPC and TFC were 1.780 ± 5.00 (µg GAE/g) and 322.91 ± 0.98 (µg QE/g), respectively. These results are lower than those found by Adisakwattane et al., who reported total phenolics, flavonoids, 45.21 ± 0.96 mg GAE/g extract, and 15.39 ± 0.58 mg catechin equivalents/g extract [34].

The antioxidant activity of *M. oleifera* leaf infusion, as measured by the DPPH free radical scavenging method, shows an ability to reduce these free radicals, which was stronger than the positive standard used, BHT. The IC_50_ of infusion and BHT were 8.19 ± 0.005 µg/mL and 11.60 ± 0.30 µg/mL, respectively. The antioxidant activity of *M. oleifera* leaf extract in this study was robust, which falls in line with reports by several researchers and other published studies [35]. Potency is closely related to the phenolic and flavonoid content [36].

**Figure 2:**
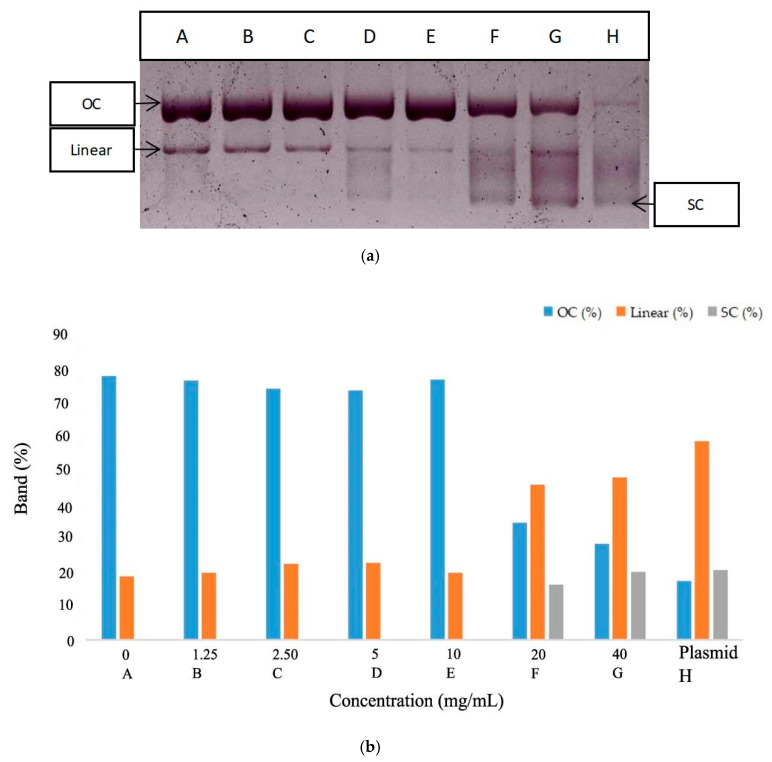
Electrophoretic monitoring of topological structure changes of the plasmid DNA (pBR322) induced by *M. oleifera* leaves infusion (**a**). Concentration-dependent inhibitory effects of *M. oleifera* leaves infusion against DNA damage expressed in % Band (**b**). Note: The letters A–H indicate the leaf infusion concentration (see Table 2).

Antioxidant compounds have an important role in protecting DNA from damage. DNA damage is usually caused by reactive oxygen species (ROS) [37,38]. *M. oleifera* leaf infusion was tested for its ability to protect DNA from the damage caused by oxidative stress. The approach used in this study was DNA damage induced by OH radicals obtained from the Fenton reaction. The pBR322 plasmid DNA was initially double-stranded. The current conformation is a supercoil (SC), where the electrophoretic mobility is high. When OH radicals bind to DNA, the DNA strands break. The disconnection of DNA results in an open-loop conformation (open circular OC) with low electrophoretic mobility. The two forms can be separated by agarose gel electrophoresis [39,40]. The infusion showed its ability to protect DNA at a concentration of 20 mg/mL, while this ability increased at a concentration of 40 mg/mL. The OC conformation decreased by 44.9% at a concentration of 20 mg/mL and 56.8% at concentration of 40 mg/mL (Table 2, Figure 2). The protective activity was strong because it restored the conformational condition, almost matching the untreated plasmid.

The SARS-CoV-2 genome consists of about 30,000 nucleotides that code for several structural proteins. The structural proteins encoded are glycosylated spike proteins (S), envelope proteins (E), membrane proteins (M), and nucleocapsid proteins (N). Several nonstructural proteins are also encoded by these nucleotides, namely nsp1 to nsp16, RNA-dependent RNA polymerase (RdRp), coronavirus Mpro, and papain-like protease (PLpro) [41].

The interactions between the antiviral compounds from *M. oleifera* leaves and some of these structural proteins have been predicted through in silico analysis using quantum chemical, molecular docking, and dynamic methods. Several nonstructural proteins were analyzed, such as nsp-9, nsp-10, and Mpro [42,43]. The flavonoids in *M. oleifera* leaves are predicted to be used as inhibitors of COVID-19 virus infection. One of the important enzymes that play a role in the life cycle of SARS-CoV-2 is Mpro. Mpro plays a major role in the viral replication process. Mpro is interesting in terms of its use as a target so that the virus replication process can be inhibited [44].

Molecular docking was performed using a grid-based technique from AutoDock Vina. Eight ligands were attached to MPro SARS-CoV-2. The results of the experiment demonstrated a strong interaction between the potent active compound and the Mpro (PDB ID 6lu7) of SARS-CoV-2. The docking results showed various modes concerning the interaction of the protein-active compound. This can be seen in the docking score (binding energy). The lowest binding energy is considered to be the most stable ligand. The lowest binding energy for all compounds is summarized in Table 3. Specific amino acid interactions that play an essential role in the protein-active compound interactions can be observed.

The table shows the binding affinity of the ligand with Mpro SARS-CoV-2 ranging from −5.1 to −8.2 kcal/mol. Among all compounds, quercetin-3′-O-glucoside showed the highest binding affinity to Mpro. These results indicate that all selected ligands exhibit good binding affinity with our target molecules. Remdesivir was chosen as a comparison because it has shown the ability to shorten the recovery time and reduce the incidence of respiratory tract infections in adults with COVID-19 [44].

The docking results were visualized to determine the interactions and binding mode of the bioactive compound–protein complexes (Table 4). Tahir ul Qamar et al. reported that the binding site area of the active site is located on Cys145 and His41 [45], and that the ligands will inhibit the performance of the receptor when the ligand is bound to the receptor’s binding site [46]. The interaction of the bioactive compounds and Mpro is shown in Table 4. The docking results showed an interaction between the bioactive compounds with Cys145 and His41. Gentiatibetine and remdesivir both bonded to one active site, His41, in the form of pi-donor hydrogen and pi-cation, respectively.

Hydrogen bonds affect the strength of the bonds between ligands and amino acid residues; the more hydrogen bonds that occur, the stronger and more stable the bond [47]. In this study, each bioactive compound has a different number of hydrogen bonds and is located in different amino acid residues. Quercetin-3′-O-glucoside has two hydrogen bonds with Mpro on the amino acid residue Phe140 and Glu166. Undulatoside A has three hydrogen bonds with Mpro, which are at residues His163, Ser144, and Cys145. Kaempferol-3-O-neohesperidoside has two hydrogen bonds with Mpro at the amino acid residues Glu166 and Gly143. Kaempferol has one hydrogen bond with the amino acid residue Leu141. Remdesivir has one hydrogen bond with His164.

**Figure 3:**
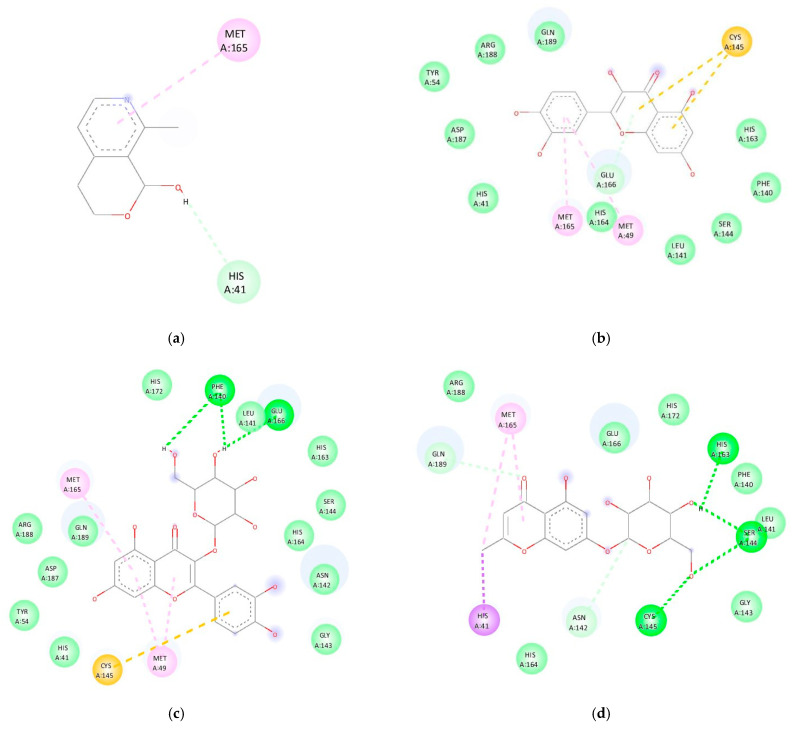

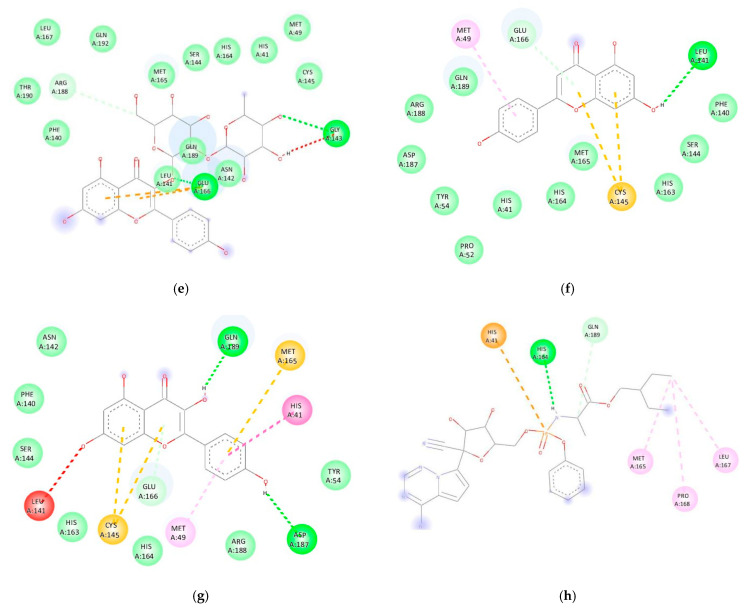
Results of Mpro docking with several active compounds in *M. oleifera* leaf infusion. (**a**) Mpro-gentiatibetine; (**b**) Mpro-quercetin; (**c**) Mpro-Quercetin-3-O-glucoside; (**d**) Mpro-Undulatoside A; (**e**) Mpro-Kaemferol-3-O-neopiroside; (**f**) Mpro-apigenin; (**g**) Mpro-Kaempferol; (**h**) Mpro-Remdesivir.

Pi-sigma and pi-alkyl bonds cause hydrophobic interactions. This hydrophobic interaction may support the inhibition of receptor action so that it can be used to design specific inhibitors. In this study, it was found that each of the bioactive compounds had hydrophobic interactions, except apigenin (Table 4, Figure 3). Pi-cation and pi-sulfur interactions increase the binding affinity of the ligand to the receptor. van der Waals forces also contribute to inhibiting the action of target receptors, although this is weaker than the hydrogen bond [47]. Each active compound exhibited the van der Waals force, except remdesivir and gentiatibetine (Table 4).

All of the above results indicate that the ligand binds to form a stable complex with the target protein (Mpro). These results can be compared to the mechanism of action between the N3 inhibitor and Mpro [48]. Hence, it can be concluded that our preferred ligand may have antiviral properties. *M. oleifera* leaves have a high flavonoid content. Flavonoids are compounds that have antiviral abilities. Most of the active compounds in the *M. oleifera* leaves that have been identified showed inhibitory potential relative to Mpro and in comparison to hydroxychloroquine. Some of these compounds include kaempferol (−7.8 Kcal/mol), myricetin (−7.7 Kcal/mol), quercetin (−7.5 Kcal/mol), ellagic acid (−7.3 Kcal/mol), epicatechin (−7.0 Kcal/mol), caffeic acid (−5.6 Kcal/mol), and gallic acid (−5.5 Kcal/mol) [49].

In addition, the most active compounds from the plants studied showed that flavonoids, ellagic acid, and apigenin were proven (in silico) to have remarkable potential as new drug candidates. This compound was able to interact with nsp-9 and nsp-10 SARS-CoV-2 with the highest binding affinities of −7.1 and −6.5 Kcal/mol against nsp-9, and −6.9 and −7.1 Kcal/mol against nsp-10 [48]. These results can be compared with several antiviral drugs used as anti-COVID-19 proteases, such as oseltamivir, ritonavir, remdesivir, Ribavirin, favipiravir, chloroquine, and hydroxychloroquine [40].

## 3. Materials and Methods

### 3.1. Preparation of M. oleifera Leaf Infusion

*M. oleifera* leaf powder was obtained from CV. Kebonqta Mubarak, South Tangerang, Indonesia. An amount of 10 g of *M. oleifera* leaf powder was dissolved in 200 mL of distilled water and then boiled at 90 °C for 20 min. After being filtered, the filtrate was analyzed for chemical content using liquid chromatography-mass spectrometry (LC-MS), measuring for its antioxidant activity and tested for its ability to prevent DNA damage.

### 3.2. Total Phenolic Content

Folin-Ciocalteau reagent was used to measure the total phenolic content. Gallic acid was used as the standard. A total of 0.5 mL of *M. oleifera* leaf infusion was added to 2.5 mL of Folin-Ciocalteu 10% reagent. Incubation was carried out for 10 min. A total of 2.5 mL Na_2_CO_3_ 75 g/L was added to the mixture. The mixture was incubated for two hours at room temperature. The absorbance was measured at 765 nm and compared with a blank solution containing only solvent (500 µL). Total phenolic content was calculated as gallic acid equivalent (GAE) from the standard curve and expressed as GAE/g dry mass [50].

### 3.3. Total Flavonoid Content

The aluminum chloride colorimetric method was used to determine the total flavonoid content using quercetin as a standard solution. The standard solution of quercetin (50 mg in 1 mL of 95% ethanol) was diluted to obtain various concentrations to prepare the standard curves. A total of 0.5 mL of the standard solution was diluted with 1.5 mL of 95% ethanol, and then mixed with 0.1 mL of 10% aluminum chloride, then 0.1 mL of 1 M sodium acetate and 2.8 mL of distilled water were added. Incubation was carried out at room temperature for 30 min. The absorbance was measured at 415 nm with a Biochrom Libra S22 spectrophotometer. The same procedure was carried out on the sample by replacing the standard solution with *M. oleifera* leaf infusion [51].

### 3.4. Phytochemical Analysis Using Liquid Chromatography-Mass Spectrometry (LC-MS)

LCMS/MS-QTQF (Waters) was used to analyze the active substances in *M. oleifera* leaf infusion. TOF MSE was used as the mode of operation. It was equipped by an ESI electrospray ionization source with positive and negative ion modes. The C18 was used as the column. Formic acid 0.1% in acetonitrile and formic acid 0.1% in aquabidest were used as the mobile phase. The total flow rate was 0.6 mL/min. A total of 0.5 g of the sample was dissolved in 10 mL of methanol then homogenized in the ultrasonicator for 30 min. Then, it was filtered using a 0.22 m GHP/PTFE membrane filter. An amount of 10 microliters of sample was injected. UNIFI software, which has a mass spectrum library of natural active substances from the Waters database, was used in the screening process to detect the active substances in samples. The sample mass spectrum identified and matched with the mass spectrum in the library was considered as the active compound. Identified compounds had to meet the following criteria: analyte reading mass error ≤ 5 ppm error, Isotope match MZ RMS ≤ 6, analyte intensity ≥ 300, and one fraction with a brake value < 4 in the fragment elucidation system.

### 3.5. Antioxidant Activity with DPPH Radical Scavenging

The free radical 2,2-diphenyl-1-picrylhydrazyl (DPPH) (Sigma Aldrich) was used to measure the antioxidant activity of *M. oleifera* leaf infusion. Antioxidant activity was obtained as a factor of the ability to extinguish free radicals. A total of 500 µL extracts with different concentrations were reacted with 1500 µL DPPH 150 µM in methanol absolute. The mixture was incubated for 30 min in the darkroom. The mixture absorbance was measured at 517 nm using a spectrophotometer (Biochrome Libra S22). The radical quenching ability of DPPH was calculated using the following formula. A standard antioxidant, butylated hydroxytoluene (BHT), was used as a reference [52].

### 3.6. DNA Protection Activity Assay

Plasmid DNA pBR322 (NEB) was used as a model to evaluate the antioxidant protective effect against DNA damage caused by free radicals. The free radical OH- was produced by the Fenton reaction. The transformation of the plasmid DNA pBR322 from supercoiled form to the open-circular and linear forms was used as an index of DNA damage [53]. The reaction mixture (15 µL) contained 5 µL of phosphate buffer saline (PBS, 10 mM, pH 7.4), 1 µL of plasmid DNA (0.5 g), 5 µL of sample, 2 µL of 1 mM FeSO_4_, and 2 µL of 1 mM H_2_O_2_. The mixture was incubated at 37 °C for 30 min. After 30 min of incubation, 2 µL of loading dye (Geneaid) was added (10 mM Tris-HCl pH 7.6, 60 mM EDTA 0.1% bromophenol blue, 0.1% xylene cyanole FF, 50% glycerol) to stop the reaction. After that, the solution mixture was electrophoresed on a 0.85% agarose gel containing 0.5 µL of gelred [54].

### 3.7. In-Silico Study of the Active Compound Infusion of M. oleifera Leaves with COVID-19 Main Protease

The target of the active compound in Moringa leaf infusion is a nonstructural protein in the SARS-CoV-2 virus, namely Mpro. All docking experiments used Discovery Studio v21.1 and Pyrx 0.8 [16]. The Mpro protein (coronavirus main protease in complex with an inhibitor N3) structure was taken from the Protein Data Bank (ID 6lu7). All ligand structures were obtained from PubChem. The chemical structure of apigenin-6-C-glucosylglucoside and kaempferol Kaempferol 3-α-L-dirhamnosyl-(1 → 4)-β-D-glucopyranoside could not be found in PubChem, so the basic structures of apigenin and kaempferol were used in this docking.

Protein preparation was done using Discovery Studio v21.1., ligand preparation using Pyrx 0.8. Ligands were converted into the most stable structure energetically using energy minimization. The ligand and protein molecules were converted to a readable file format (pdbqt) using Pyrx 0.8. Docking was done on the active site of the main protease (Mpro) with N3 removed. Discovery studio was used to find the active site position to determine the X, Y, and Z values. The values obtained were used to create grid boxes in the docking process with Pyrx. A grid box was used to cover the entire active site of the protein structure. This was carried out to find the possible binding of protein-ligand. All dockings were presented by Pyrx 0.8. The final visualization of the anchored structure was carried out by Discovery Studio Visualizer v21.1 [54].

### 3.8. Data Analysis

Linear regression was used to analyze the antioxidant activity. Data were presented in mean ± standard deviation (SD). Azzure software was used to analyze the antimutagenic activity.

## 4. Conclusions

Referring to the purpose of this study, it can be concluded that the bioactive compounds found in *M. oleifera* leaf powder infusion include alkaloid, flavonoid, and chromone derivative groups. Gentiabatine was included in the alkaloid group. Flavonoids were found in glycoside states, namely quercetin 3-O-glucoside, kaempferol 3-o-neohesperidoside, and kaempferol Kaempferol 3-α-L-dirhamnosyl-(1 → 4)-β-D-glucopyranoside. The flavonoid derivative of chromone was undulatoside A.

The antioxidant activity of *M. oleifera* leaf infusion was determined by the presence of the bioactive compounds mentioned above. The antioxidant activity of *M. oleifera* leaf powder is stronger than BHT. The criteria for protective activity against DNA were strongly met.

The bioactive compounds, gentiabatine, quercetin, quercetin 3-O-glucoside, kaempferol 3-o-neohesperidoside, and undulatoside A are potential candidates for anti-COVID-19 treatments.

## 5. Recommendations

In vivo studies are needed to determine the protective activity of *M. oleifera* extract towards DNA, concerning, for example, cancer and some other degenerative diseases. In vitro and in vivo antiviral studies are necessary to confirm these findings.

## Figures and Tables

**Table 1:** LC-MS phytochemical analysis.

No	Identified Compounds	Ionization Mode	RT	MZ	Molecular Formula	Response
Alkaloid						
1	Gentiatibetine	positive	3.37	77, 103, 120	C_9_H_11_NO_2_	12.820
Flavonoid						
2	Quercetin	positive	8.48	303, 304, 487	C_15_H_10_O_7_	22.083
3	Kaempferol 3-α-L-dirhamnosyl-(1 → 4)-β-D-glucopyranoside	positive	6.55	457, 495, 633	C_27_H_30_O_15_	7.934
4	Apigenin-6-C- glucosylglucoside	negative	6.58	353, 593, 646	C_27_H_30_O_15_	24.929
5	Quercetin-3′-O-glucoside	negative	8.53	271, 300, 463	C_21_H_20_O_12_	98.283
6	Undulatoside A	negative	5.12	173, 191, 353	C_16_H_18_O_9_	51.556
7	Kaempferol-3-Oneohesperidoside	negative	9.24	301, 593, 607	C_27_H_30_O_15_	12.952

**Table 2:** Antimutagenic analysis.

Code	Treatment	Nick (%)	Linear (%)	SC (%)
A	Plasmid + H_2_O_2_ + Fe_2_SO_4_	80.6	19.4	
B	1.25 mg/mL infusion + Plasmid + H_2_O_2_ + Fe_2_SO_4_	79.4	20.6	
C	2.5 mg/mL infusion + Plasmid + H_2_O_2_ + Fe_2_SO_4_	76.8	23.2	
D	5 mg/mL infusion + Plasmid + H_2_O_2_ + Fe_2_SO_4_	76.4	23.5	
E	10 mg/mL infusion + Plasmid + H_2_O_2_ + Fe_2_SO_4_	79.6	20.4	
F	20 mg/mL infusion + Plasmid + H_2_O_2_ + Fe_2_SO_4_	35.7	47.5	16.8
G	40 mg/mL infusion + Plasmid + H_2_O_2_ + Fe_2_SO_4_	29.5	49.6	20.8
H	Non treated Plasmid	17.9	60.6	21.4

**Table 3:** The best binding energy scores of the active compounds and the target proteins of Mpro.

No	Active Compound	Mpro (6lu7) (kcal/mol)	rmsd/ub	rmsd/lb
1	Gentiatibetine	−5.1	0.00	0.00
2	Quercetin	−7.5	0.00	0.00
3	Quercetin-3′-O-glucoside	−8.2	0.00	0.00
4	Undulatoside A	−7.7	0.00	0.00
5	Kaempferol-3-O-neohesperidoside	−5.7	0.00	0.00
6	Apigenin	−7.8	0.00	0.00
7	Kaempferol	−7.8	0.00	0.00
8	Remdesivir	−7.3	0.00	0.00

**Table 4:** The interaction between Mpro and the bioactive compounds.

No	Active Compound	Interaction	Amino Acid Residues
1	Gentiatibetine	pi-donor hydrogen	**His41**
		pi-alkyl	Met165
2	Quercetin	pi-sulfur	**Cys145**
		pi-alkyl	Met49
		pi-donor hydrogen	Glu166
		van der Waals	His163, Phe140, Ser144, Leu141, His164, **His41**, Asp187, Tyr54, Arg188, and Gln189
3	Quercetin-3′-O-glucoside	conventional hydrogen	Phe140 and Glu166
		van der Waals	His172, Leu141, His163, Ser144, His164, Asn142, Gly143, Arg188, Gln189, Asp187, Tyr54, **His41**
		pi-sulphur	**Cys145**
		pi-alkyl	Met49 and Met165
4	Undulatoside A	pi-sigma	**His41**
		conventional hydrogen	His163, Ser144, **Cys145**
		carbon hydrogen	Gln189, Asn142
		van der Waals	Arg188, His164, Glu166, His172, Phe140, Leu141 and Gly143
5	Kaempferol-3-O-neohesperidoside	conventional hydrogen	Glu166 and Gly143
		pi-anion	Gly143
		unfavorable donor-donor	Gly143
		carbon-hydrogen	Arg188
		van der Waals	Phe140, Thr190, Leu167, Gln192, Met165, Leu141, Gln149, Asn142, Ser144, His164, **His41**, Met49, **Cys145**
		pi-alkyl	Met49
6	Apigenin	pi-sulphur	**Cys145**
		hydrogen-donor pi	Glu166
		van der Waals force	Gln189, Arg188, Asp187, Tyr54, Pro52, **His41**, His164, Met165, Leu141, Phe140, His163, and Ser144
7	Kaempferol	conventional hydrogen bond	Gln189 and Asp187
		pi-donor hydrogen	Glu166
		pi-sulphur	**Cys145**, Met165
		pi-alkyl	Met49
		pi-pi stacked	**His-41**
		Unfavorable acceptor-acceptor	Leu141
		van der Waals	Asn142, Phe140, Ser144, His163, His164, Arg188, Tyr54
8	Remdesivir	pi-alkyl	Met165, Pro168, Leu167
		carbon-hydrogen	Gln189
		pi-cation	**His41**
		hydrogen	His164

Note: amino acid in bold means the amino acid in the binding site.

## Data Availability

The data presented in this work are available in the article.
